# Defined Microbiota Modulates Host Metabolome and Skeletal Adaptation to Diet‐Induced Obesity

**DOI:** 10.1096/fj.202600564RR

**Published:** 2026-04-21

**Authors:** Melanie Cristine Scalise, Mathieu Simon, Jasmin Bernhardt, Ora Trümpi, Timm Hettich, Stefan Gaugler, Nikola Saulacic, Benjamin Gantenbein, Philippe Zysset, Maria Luisa Balmer

**Affiliations:** ^1^ Institute for Infectious Diseases University of Bern Bern Switzerland; ^2^ Diabetes Center Berne Bern Switzerland; ^3^ Graduate School for Cellular and Biomedical Sciences University of Bern Bern Switzerland; ^4^ ARTORG Center for Biomedical Engineering Research University of Bern Bern Switzerland; ^5^ School of Life Sciences FHNW Institute for Chemistry and Bioanalytics Muttenz Switzerland; ^6^ Clinic for Cranio‐Maxillofacial Surgery, Department for BioMedical Research (DBMR) University of Bern Bern Switzerland; ^7^ Bone & Joint Program, Department for BioMedical Research (DBMR) University of Bern Bern Switzerland; ^8^ Department of Orthopaedic Surgery & Traumatology Inselspital, Bern University Hospital, University of Bern Bern Switzerland

## Abstract

The gut microbiota is increasingly recognized as a regulator of host metabolism and bone physiology. However, how microbial colonization integrates systemic metabolic cues with skeletal remodeling under metabolic stress remains unclear. We used germ‐free (GF) and gnotobiotic C57BL/6J mice colonized with the defined 12‐member Oligo‐Mouse‐Microbiota (Oligo‐MM^12^) to dissect microbiota‐dependent bone adaptation during high‐fat diet (HFD)–induced obesity. Micro‐CT analysis revealed that only colonized mice exhibited structural adaptations, namely increased cortical thickness and trabecular area, in response to HFD, whereas GF mice failed to remodel their skeleton despite broadly comparable weight gain trajectories and adiposity. Serum metabolomics uncovered distinct microbiota‐specific metabolic signatures. GF mice accumulated bone‐relevant metabolites including lysine, uridine, DHA, and pyruvate, suggesting altered systemic handling of bone‐relevant metabolites, whereas colonized mice displayed reduced circulating levels associated with skeletal remodeling. These metabolic patterns correlated with reduced β‐CTX levels in colonized mice, indicative of microbiota‐mediated suppression of bone resorption. Our findings identify the gut microbiota as a key determinant of skeletal adaptation to diet‐induced obesity, presumably acting through systemic metabolic reprogramming and modulation of bone turnover. The defined‐microbiota mouse model provides a powerful framework to disentangle the gut–bone axis at a systems and metabolic level.

## Graphical abstract

Germ‐free mice exposed to high‐fat diet accumulate circulating bone‐relevant metabolites but fail to undergo adaptive skeletal remodeling. In contrast, mice colonized with a defined Oligo‐MM^12^ microbiota display altered systemic metabolite profiles associated with appropriate trabecular and cortical bone adaptation. These findings identify the gut microbiota as a key determinant linking metabolic state to skeletal remodeling during diet‐induced obesity.

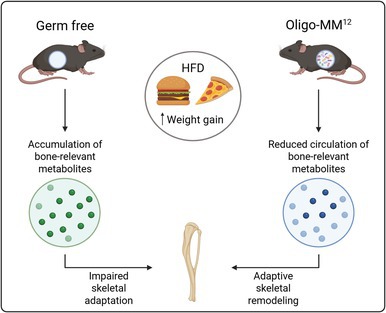

## 1 Introduction

Bone is a dynamic tissue that undergoes continuous remodeling, a process regulated by systemic factors such as mechanical load, hormonal signaling, calcium/phosphate metabolism and dietary intake. Among these factors, obesity has been shown to influence bone structure, leading to changes in both cortical and trabecular bone. While obesity is traditionally associated with increased bone mass due to higher mechanical loading, its effect on bone quality seems to be controversial among species.

In humans, osteodensitometry measurements such as bone mineral density (BMD) and bone volume fraction (BV/TV) often do not show significant differences between obese and nonobese individuals [1, 2, 3, 4, 5]. Despite similar or even increased BMD, obesity has been linked to a higher risk of fractures. This might be explained by a higher propensity to fall and lower ability to reduce the impact during falls. However, excess adiposity may also negatively impact bone microarchitecture and strength beyond what is captured by densitometry alone [6, 7, 8].

Studies in mice have demonstrated that obesity induces distinct skeletal adaptations, including increased bone mass in response to mechanical loading but also alterations in trabecular and cortical bone architecture that may compromise overall bone mechanical competence [3, 9, 10]. The precise mechanisms underlying these obesity‐induced skeletal changes remain poorly understood, particularly regarding the role of the gut microbiota and metabolic factors in bone remodeling.

An emerging area of research has highlighted the role of the gut microbiota in skeletal homeostasis. The gut microbiota, composed of trillions of microorganisms residing in the gastrointestinal tract, influences various physiological processes, including immune regulation, metabolism, and nutrient absorption [11, 12, 13, 14, 15].

The gut microbiota plays a crucial role in bone health through several mechanisms. First, microbial metabolites such as short‐chain fatty acids (SCFAs) enhance calcium absorption and regulate osteoclast and osteoblast activity, directly affecting bone turnover [16]. Second, the microbiota modulates immune responses, influencing cytokine production and systemic inflammation, both of which are critical determinants of bone remodeling [17]. Third, alterations in gut microbiota composition, known as dysbiosis, have been associated with metabolic disorders, including obesity and osteoporosis, further linking microbial communities to skeletal integrity [18]. Studies in germ‐free (GF) mice have demonstrated that the absence of microbiota leads to altered bone microarchitecture with increased bone mass and reduced osteoclast abundance as compared to conventionally‐colonized mice, suggesting that microbial signals are necessary for normal bone development and adaptation [19]. Additionally, studies have demonstrated that microbiota composition affects calcium absorption and bone turnover [20]. Recent findings suggest that certain bacterial species may prevent bone loss [21]. Despite these advances, the causal contribution of defined microbial consortia to skeletal remodeling under metabolic stress has not been directly demonstrated.

Studying host‐microbiota interactions in humans is limited by ethical constraints, dietary variability, and complex microbial ecosystems, which confound causal inference. Likewise, conventional specific pathogen‐free (SPF) mouse models harbor undefined microbiotas and inter‐individual variation, making it difficult to attribute phenotypic changes to specific microbes or functions. In contrast, the gnotobiotic mouse model used in this study features a defined and stable consortium of 12 murine intestinal strains (Oligo‐MM^12^) [22] that recapitulates key immunological and metabolic traits of SPF mice, while maintaining experimental reproducibility and microbiota control. This model provides a unique platform to dissect causal relationships between microbial signals and host physiology in vivo.

Given the microbiota's role in metabolic regulation and bone remodeling, we hypothesize that it critically mediates obesity‐induced skeletal adaptations through combined effects on systemic metabolite profiles and local remodeling processes. In this study, we used a highly controlled gnotobiotic mouse model colonized with the defined Oligo‐MM^12^ consortium to dissect how the gut microbiota shapes bone remodeling in response to high‐fat diet‐induced obesity. The hypothesis was that microbial colonization would enable proper skeletal adaptation by coupling systemic metabolic cues to local bone turnover.

## 2 Materials and Methods

### 2.1 GF and Oligo‐MM^12^ Mice

Age‐matched female germ‐free (GF) and gnotobiotic Oligo‐MM^12^ C57BL/6J mice were bred and housed at the Clean Mouse Facility Bern (CMF). The germ‐free status of all animals has been routinely monitored using culture‐based and culture‐independent methods established and validated by the CMF. Oligo‐MM^12^ mice used in this study originated from a stable breeding colony and were colonized by vertical transmission rather than by oral gavage.

Oligo‐MM^12^ mice harbor the defined 12‐member Oligo‐Mouse‐Microbiota (Oligo‐MM^12^) community originally described by Brugiroux et al. [22]. The consortium consists of: *Clostridium innocuum* I46, *Bacteroides caecimuris* I48, *Limosiactobacillus reuteri* I49, *Enterococcus faecalis* KB1, *Acutalibacter muris* KB18, *Bifidobacterium animalis* subsp. *animalis* YL2, *Muribaculum intestinale* YL27, *Flavonifractor plautii* YL31, *Enterocloster clostridioformis* YL32, *Akkermansia muciniphila* YL44, *Turicimonas muris* YL45, and *Blautia pseudococcoides* YL58.

The successful establishment and stability of all 12 strains have been previously validated by metagenomic sequencing ([22, 23, 24, 25, 26, 27, 28, 29]) and were confirmed again for this study (Figure S1A).

The original anaerobic cultivation, equal cell‐count adjustment, cryopreservation, and inoculation procedures for generating Oligo‐MM^12^ mice have been described in detail previously [22, 23]. The colony is stably maintained in flexible‐film isolators under strict gnotobiotic conditions and routinely monitored to ensure preservation of the defined community structure [28, 30].

Mice were group‐housed (five animals per cage) under standardized environmental conditions. Allocation to experimental groups was performed randomly within each microbiota condition.

All groups of mice were fed an autoclaved chow diet (Kliba‐Nafag 3307, Granovit, CH) *ad libitum* from birth until diet switch to an experimental diet. Animal work was carried out in compliance with the guidelines of the Swiss Federal Veterinary Office and was approved by the Cantonal Ethical Committee under animal experimentation permission BE48/2022.

### 2.2 Firmicutes Mice

Gnotobiotic mice harboring a reduced five‐member Firmicutes consortium were generated by *de novo* colonization of germ‐free C57BL/6J mice. The consortium consisted of *Enterococcus faecalis* KB1, *Flavonifractor plautii* YL31, *Enterocloster clostridioformis* YL32, *Clostridium innocuum* I46, and *Blautia pseudococcoides* YL58.

Individual strains were cultivated anaerobically at 36°C in pre‐reduced media as previously described for Oligo‐MM^12^ strains. Cultures were adjusted to comparable optical densities, pooled in equal proportions, and used immediately for colonization. Germ‐free recipient mice were colonized by oral gavage with 100 μL of the bacterial suspension under sterile conditions. To enhance colonization efficiency, gavage was performed twice (Day 0 and Day 3).

Colonized animals were subsequently maintained under gnotobiotic conditions in flexible‐film isolators and allowed to stabilize prior to diet intervention. Community composition and successful engraftment of the five strains were verified by 16S rRNA gene sequencing (Figure S2A).

### 2.3 Experimental Diets

**FIGURE 1:**
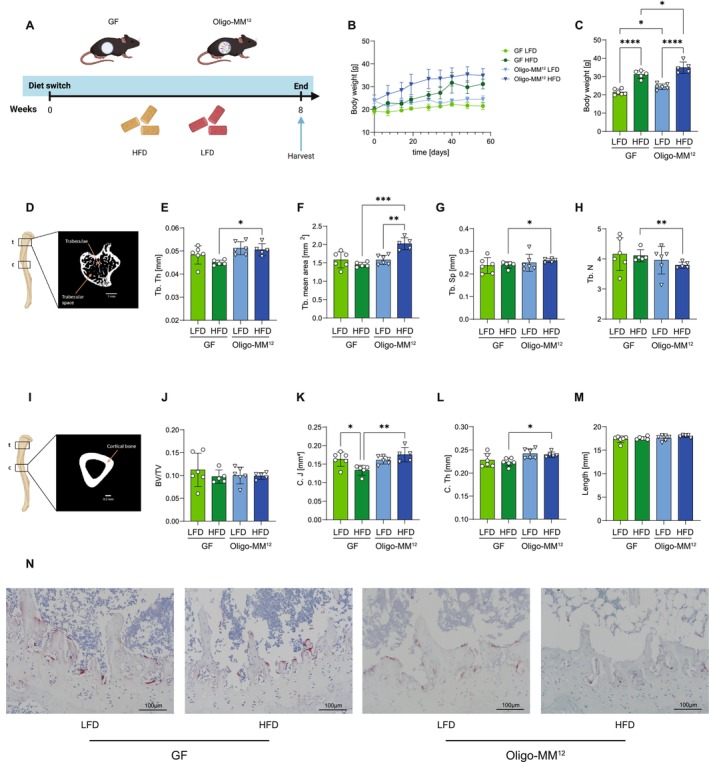
Microbiota modulates bone phenotype during high‐fat‐diet‐induced obesity in gnotobiotic mice. (A) Experimental design: Germ‐free (GF) mice or mice colonized with the simplified defined microbiota Oligo‐MM^12^ were fed a high‐fat diet (HFD) or low‐fat diet (LFD) for 8 weeks, followed by tissue harvest and analysis. Femoral trabecular and cortical bone architecture were assessed by micro‐CT. Created with BioRender.com. (B) Body weight trajectory over the 8‐week period. (C) Final body weight at harvest. (D) Representative micro‐CT image of the tibial trabecular region and schematic of measurement site. (E–H) Trabecular bone parameters assessed by micro‐CT: (E) trabecular thickness (Tb.Th), (F) trabecular mean area (Tb. mean area), (G) trabecular separation (Tb.Sp), (H) and trabecular number (Tb.N). (I) Representative micro‐CT image of the cortical tibial region and schematic of measurement site. Created with BioRender.com. (J–M) Cortical bone parameters: (J) bone volume fraction (BV/TV), (K) cortical polar moment of inertia (C.J), (L) cortical thickness (Ct.Th), (M) and tibial length. (N) Representative TRAP‐stained sections showing osteoclasts in tibiae from GF and Oligo‐MM^12^ mice fed LFD or HFD. (scale bar: 100 μm). Statistical analysis: Two‐way ANOVA with Šídák's multiple comparisons test was applied for factorial analyses (diet × microbiota). For specific two‐group comparisons in panels H and I, the non‐parametric Mann–Whitney test was used. Data are presented as mean ± SD. *p < 0.05, **p < 0.01, ***p < 0.001, ****p < 0.0001. *n* = 5–6 per group. Figures were created with BioRender.com.

At 16–19 weeks of age, mice were switched to either a high fat diet (HFD; modified rodent diet with 60 kcal% fat obtained from Research Diets, caloric content 5240 kcal/kg) or a low‐fat control diet (LFD: modified rodent diet with 10 kcal% fat from Research Diets, caloric content 3850 kcal/kg) *ad libitum*. Both diets were irradiated twice with 20–40 kGy for sterilization by the producing company and a third time before being administered to mice by Steris AG (Synergy Health Däniken AG). Mice were fed this experimental diet for 8 weeks. Body weight was monitored weekly during experimental diet administration (Figure 1A). At harvest, tibiae and fibulae were carefully dissected free of surrounding muscle tissue, wrapped in saline‐moistened gauze, and stored at −20°C until further processing.

### 2.4 DNA Extraction and 16S rRNA Gene Sequencing

Cecal contents were collected under sterile conditions and stored at −80°C until processing. Microbial DNA was extracted using the DNeasy PowerSoil Pro Kit (Qiagen, Hilden, Germany) according to the manufacturer's instructions.

For microbial community profiling, 16S rRNA gene sequencing was performed by the Next Generation Sequencing (NGS) Facility of the University of Bern using an Illumina platform. Sequencing data were processed using standard bioinformatic pipelines for quality filtering, taxonomic assignment, and calculation of relative abundances. These analyses were used to verify community composition and engraftment in Oligo‐MM^12^ and Firmicutes‐colonized mice (Figures S1A and S2A).

### 2.5 Micro‐Computed Tomography

Micro‐computed tomography (μCT) analyses were performed on the tibia after removing the fibula by using a μCT 100 scanner from Scanco Medical AG, Brütisellen, Switzerland. Tibiae were imaged with an X‐ray tube voltage of 70 kVp and a current of 85 mA (recommended to keep low for higher contrast) with an integration time of 200 ms and an aluminum filter of 0.5 mm. The field of view (FOV) and the number of projections/180° was adjusted to reach the desired voxel size. Full bone was scanned with a voxel size of 34 μm, and two regions of interest (ROIs) were selected and scanned at a voxel size of 10 μm. The first 1 mm ROI consists of the proximal part of the bone starting 0.2 mm proximal to the section where the lateral and medial part of the growth plate merged. The second 1 mm thick ROI is centered at the tibial midshaft (Figure 1B).

### 2.6 Image Processing

The μCT reconstructions of the two ROIs were used to analyze standard trabecular and cortical morphometric parameters. Bone morphometry such as cortical thickness (C. Th in mm), cortical polar moment of inertia (C.J in mm^4^), trabecular thickness (Tb. Th in mm), trabecular separation (Tb. Sp in mm), trabecular number (Tb. N), trabecular mean area (Tb. mean area in mm^2^), bone volume fraction (BV/TV), and full bone length (in mm) were evaluated using the manufacturer's software.

### 2.7 Analysis of Primary Metabolites

The metabolomics analysis was performed following a procedure described by Fiehn [31].

Blood was collected in a serum separation tube, and 30 μL of the separated serum was aliquoted from the top. The samples for the analysis were extracted with 1000 μL of an ice‐cold (−20°C) ethanol‐water mixture (3:1, v/v). The extraction solvent was degassed for 5 min under a flow of nitrogen and cooled overnight in the freezer at −20°C before sample extraction. A 5 mm stainless steel ball was placed inside the tubes with the extraction solvent, and the mixture was processed using a ball mill MM 400 (Retsch, Germany) at a vibration speed of 20 Hz for 30 s with two run cycles. The solution was further shaken at 10°C with 1400 rpm for 5 min on a Thermomixer Comfort (Eppendorf, Switzerland). Samples were centrifuged for 2 min at 14,000 rpm. The resulting 400 μL supernatant was evaporated to dryness with a GeneVac EZ‐2 Plus (StepbioS, Switzerland) at 40°C. The dry extract was redissolved in 10 μL of 20 mg/mL of O‐methylhydroxylamine hydrochloride anhydrous pyridine solution and agitated with 1400 rpm for 90 min at 30°C. For the second derivatization step, 91 μL N‐methyl‐N‐trimethylsilyltrifluoroacetamide (MSTFA) was added and shaken at 1400 rpm for 30 min at 37°C. Before derivatization, 990 μL of the MSTFA was mixed with 10 μL of fatty acid methyl esters (FAME, C8‐C16, C18‐C30) for internal retention marker index.

The analysis was performed on an Agilent 7890B gas chromatograph (GC) equipped with a cooled injection system (Gerstel, Switzerland). The GC was hyphenated with a time‐of‐flight mass spectrometer Pegasus BT (Leco, Germany). The system was equipped with an electron impact (EI) source. Chromatographic separation was achieved on an Rtx‐5 column (30 m × 0.25 mm i.d. and 0.25 μm film thickness, Restek, USA) with an integrated 10 m guard column. Helium was used as carrier gas at a constant flow rate of 1 mL/min. An aliquot of 0.5 μL was injected into a 2 mm dimpled splitless liner at an initial temperature of 50°C, which was ramped to 275°C with 12°C/s. The split vent was opened after 25 s. The oven temperature program started at 50°C (hold for 1 min), increased to 330°C at 20°C/min and held for 5 min before cooldown. The ion source operated at 70 eV. The source temperature was set to 250°C, and the transfer line was heated to 280°C. Mass spectra were acquired in scan mode between *m/z* 85–500 at 15 Hz. The GC–MS system was controlled by Leco ChromaTOF version 5.51.

The raw Leco data files were exported to netCDF and further converted to Analysis Base Files using Reifycs Abf Converter (v.1.3). Data files containing chromatographic and mass spectral data were then deconvoluted with MS‐Dial (v. 5.50) [32]. Metabolite identification was achieved using the FiehnLib mass spectral and retention index library [33]. All aligned identified and unidentified compounds were exported to a text file. Annotated compounds were manually inspected and curated in Microsoft Excel 2016 and MS Dial. A comma‐separated values (CSV) file was generated for statistical analysis. Intensities were log‐transformed and *Z*‐score normalized across samples. Principal component analysis (PCA) was performed using the scikit‐learn implementation in Python to identify global patterns and group separation. Heatmaps were generated using seaborn's clustermap with hierarchical clustering of both metabolites and samples based on Euclidean distance. For volcano plots, fold changes were calculated as log_2_(GF/Oligo‐MM^12^) means and statistical significance was assessed using two‐tailed unpaired *t*‐tests for each metabolite. Significance thresholds were set at *p* < 0.05 and |log_2_FC| > 1. Figures were created in matplotlib. *p* values were adjusted for multiple testing using the Benjamini–Hochberg false discovery rate correction.

### 2.8 Histology

Tibiae were harvested from mice, cleaned of soft tissue, and fixed in 4% formaldehyde (Grogg Chemie, Art. no. G256). Samples were decalcified in 4.13% EDTA solution for 10 weeks, dehydrated through an ascending ethanol/xylol series, embedded in paraffin, and sectioned at 5 μm using a Leica RM2255 rotary microtome.

TRAP staining solution was freshly prepared from naphthol AS‐TR phosphate disodium salt (Sigma, N6125), N,N‐dimethylformamide (Sigma, 319 937), glacial acetic acid (Emsure), sodium tartrate dihydrate (Sigma, 1.06664), and Fast Red TR salt (Sigma, 368 881). Sections were incubated in this solution at 37°C for 60 min in a water bath. After incubation, sections were counterstained with Meyer's hematoxylin for 1 s, rinsed, and mounted with Aquatex mounting medium (Sigma, 1.08562). Images were acquired using a digital microscope (VHX‐6000, Keyence, Japan).

### 2.9 Statistics

All statistical analyses were performed using GraphPad Prism version 10.5.0 (GraphPad Software). Data are presented as mean ± SD unless otherwise indicated. Normality of data distribution was assessed using the Shapiro–Wilk test.

For comparisons between two independent groups, unpaired two‐tailed Welch's *t*‐tests were applied when data were normally distributed; otherwise, the nonparametric Mann–Whitney test was used.

For experiments involving the two independent variables diet and microbiota, datasets were analyzed using ordinary two‐way analysis of variance (ANOVA), including assessment of interaction effects. Where appropriate, post hoc multiple comparisons were performed using Šídák's correction to test predefined pairwise contrasts.

A *p* value < 0.05 was considered statistically significant.

## 3 Results

### 3.1 Adaptations in Bone Microarchitecture in Response to Diet‐Induced Obesity Are Influenced by the Gut Microbiota

In order to determine the contribution of the gut microbiota in obesity‐driven bone adaptation, gnotobiotic wildtype C57BL/6 mice either germ‐free (GF) or colonized with the defined Oligo‐MM^12^ microbiota were put on a purified high‐fat diet (HFD) or matched low‐fat diet (LFD) *ad libitum* for 8 weeks. The presence and relative abundance of all 12 strains in the Oligo‐MM^12^ consortium were confirmed at the time of analysis, demonstrating stable colonization across animals as previously published [22, 23] (Figure S1A).

Whereas colonized mice exhibited higher baseline body weights as compared to GF mice at baseline, both groups increased their body weights by about 50% on HFD as compared to LFD after 8 weeks (Figure 1B,C). Consistent with previous reports in gnotobiotic models [34], food consumption was modestly increased in HFD‐fed animals, with GF mice displaying a tendency toward slightly higher intake compared to colonized counterparts. However, overall food intake did not differ significantly between microbiota conditions, indicating that differences in skeletal adaptation were not primarily driven by altered caloric consumption (Figure S1B).

To further characterize adiposity beyond total body weight, subcutaneous and visceral adipose tissue depots were quantified at harvest (Figure S1C,D). As expected, HFD feeding significantly increased both subcutaneous and visceral fat mass in GF and Oligo‐MM^12^ mice. These findings were consistent with the observed body weight gain and indicate that both microbiota groups developed comparable diet‐induced adiposity.

Overall, these results demonstrate that 8 weeks of HFD in gnotobiotic mice are sufficient to induce significant weight gain and fat accumulation.

Micro‐CT analysis revealed significant differences in trabecular bone parameters across groups. Whereas trabecular parameters on LFD were not different between GF and Oligo‐MM^12^ mice, trabecular thickness was significantly reduced in GF‐HFD mice compared to Oligo‐MM^12^ ‐HFD mice (Figure 1E). Moreover, trabecular mean area, representing the average cross‐sectional area of trabecular bone structures in the region of interest, was significantly increased in Oligo‐MM^12^ ‐HFD mice as compared to their lean Oligo‐MM^12^ ‐LFD counterparts as well as GF‐HFD mice (Figure 1F). In addition, trabecular separation was significantly increased in Oligo‐MM^12^ ‐HFD mice compared to GF‐HFD, which was accompanied by a corresponding significant decrease in trabecular number (Figure 1G,H). Of note, bone volume fraction remained unchanged across all groups (Figure 1J).

The cortical polar moment of inertia, a key biomechanical parameter indicating a bone's resistance to torsional and bending forces, was significantly decreased in GF‐HFD mice as compared to GF‐LFD mice. Furthermore, Oligo‐MM^12^ ‐HFD mice exhibited a significant increase in cortical polar moment of inertia compared to GF‐HFD mice (Figure 1K). Similarly, cortical thickness was significantly increased in Oligo‐MM^12^ ‐HFD mice compared to GF‐HFD mice (Figure 1L). Importantly, bone length (Figure 1M) remained unchanged across all tested groups.

These results indicate that the presence of a defined gut microbiota is associated with enhanced trabecular and cortical skeletal adaptation in response to HFD‐induced weight gain.

Histological assessment of bone remodeling in GF and Oligo‐MM^12^ colonized mice in response to HFD challenge showed a trend toward more numerous and intensely TRAP‐positive osteoclasts along the trabecular bone surfaces of GF mice, whereas Oligo‐MM^12^ ‐colonized mice displayed fewer TRAP‐positive cells. In addition, Oligo‐MM^12^ colonized HFD‐fed mice showed marked fat accumulation within the bone marrow cavity compared to GF mice. (Figure 1N).

### 3.2 Serum Bone Markers and Metabolites Are Altered in GF Versus Colonized Animals in Response to HFD

**FIGURE 2:**
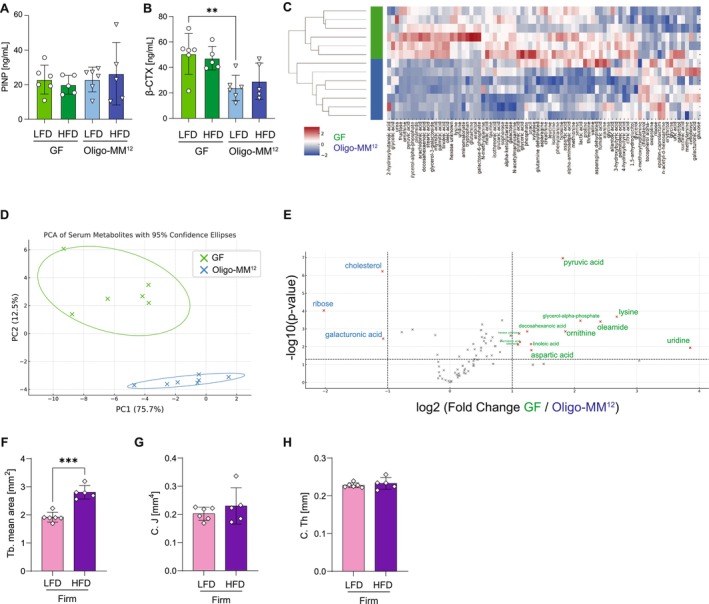
Microbiota‐dependent differences in serum bone turnover markers, systemic metabolite profiles, and skeletal adaptation in Firmicutes‐colonized mice. (A) Serum levels of procollagen type I N‐terminal propeptide (P1NP), a biochemical marker of bone formation. (B) Serum levels of C‐terminal telopeptide of type I collagen (β‐CTX), a biochemical marker of bone resorption. (C) Heatmap of relative abundance of serum metabolites from untargeted metabolomics profiling. Each row represents a mouse fed a high‐fat diet (HFD), and each column a metabolite. (D) Principal component analysis (PCA) of serum metabolomic profiles. Each point represents one mouse fed a HFD, colored by microbiota status. (E) Volcano plot illustrating metabolite abundance differences between GF and Oligo‐MM^12^‐colonized mice. Dots represent individual metabolites; significantly different metabolites are highlighted based on a threshold of *p* < 0.05 and fold change > ±1. Metabolites enriched in GF are shown in green, and those enriched in Oligo‐MM^12^ mice in blue. (F) Trabecular mean area (Tb. mean area) in mice colonized with a reduced five‐member Firmicutes consortium (Firm) fed LFD or HFD. (G) Cortical polar moment of inertia (C.J) in Firmicutes‐colonized mice. (H) Cortical thickness (C.Th) in Firmicutes‐colonized mice. Data are presented as mean ± SD. Statistical analysis: Two‐way ANOVA with Šídák's multiple comparisons test for panels (A, B); unpaired two‐tailed *t*‐tests for panels (E–H). *p < 0.05, **p < 0.01, ***p < 0.001. *n* = 5–6.

To biochemically assess bone remodeling in the presence or absence of a microbiota during HFD exposure, serum levels of procollagen type I N‐terminal propeptide (P1NP) and C‐terminal telopeptide of type I collagen (β‐CTX) across all experimental groups were measured. While P1NP, a marker of bone formation, was unchanged (Figure 2A), β‐CTX, a marker of bone resorption, was reduced in Oligo‐MM^12^ ‐colonized mice fed a LFD, consistent with the increased trabecular and cortical bone mass observed by micro‐CT. A similar, though non‐significant, trend was noted in colonized mice fed a HFD (Figure 2B).

To gain mechanistic insight into microbiota‐dependent metabolic differences of skeletal remodeling, serum metabolomics was performed in GF and colonized HFD‐fed mice. Principal component analysis (PCA) and hierarchical clustering revealed clear separation between microbiota conditions, indicating distinct systemic metabolic signatures (Figure 2C,D). Volcano plot analysis identified 15 metabolites differentially abundant between groups (Figure 2E), of which 12 metabolites were upregulated in GF mice and three in Oligo‐MM^12^ animals. These metabolites spanned multiple metabolic pathways, including amino acid, fatty acid, carbohydrate, nucleotide, and steroid metabolism (Table 1).

**TABLE 1:** KEGG grouped metabolites.

KEGG category	Metabolite	Direction in GF vs. Oligo‐MM ^12^
Amino acid metabolism	Lysine	↑ GF
	Aspartic acid	↑ GF
	Ornithine	↑ GF
Lipid metabolism	Arachidonic acid	↑ GF
	DHA	↑ GF
	Linoleic acid	↑ GF
	Oleic acid	↑ GF
	Oleamide	↑ GF
Carbohydrate metabolism	Pyruvic acid	↑ GF
	Glycerol‐α‐phosphate	↑ GF
	Hexose unknown	↑ GF
Nucleotide metabolism	Uridine	↑ GF
	Ribose	↑ Oligo‐MM ^12^
	Galacturonic acid	↑ Oligo‐MM ^12^
Steroid metabolism	Cholesterol	↑ Oligo‐MM ^12^

*Note:* KEGG pathway classification of differentially abundant serum metabolites in GF versus Oligo‐MM^12^‐colonized mice. Metabolites identified through untargeted serum metabolomics were grouped according to KEGG metabolic pathways. The table lists representative metabolites and their relative abundance direction between germ‐free (GF) and Oligo‐MM^12^‐colonized mice on HFD. Upward arrows (↑) indicate higher abundance in the indicated group. GF mice exhibited increased levels of multiple metabolites associated with amino acid, lipid, and carbohydrate metabolism, while Oligo‐MM^12^‐mice showed enrichment in select nucleotide and steroid metabolites.

Among these, GF mice exhibited increased circulating levels of metabolites previously implicated in bone metabolism, including lysine, uridine, docosahexaenoic acid (DHA), linoleic acid, and pyruvate (Figure S1E–I). In contrast, ribose levels were higher in colonized mice (Figure S1J).

Together, these data indicate that microbial colonization is associated with distinct systemic metabolic profiles in response to HFD.

### 3.3 A Reduced Firmicutes‐Based Microbiota Is Sufficient to Support Skeletal Adaptation During HFD

To further refine the causal contribution of microbial colonization to skeletal remodeling, we analyzed an independent cohort of mice colonized with a reduced Firmicutes‐dominated consortium. Sequencing‐based analysis confirmed the presence and stable relative abundance of the Firmicutes strains in colonized animals, demonstrating successful engraftment of the reduced microbial community (Figure S2A). Similar to Oligo‐MM^12^‐colonized animals, Firmicutes‐colonized mice exhibited significant body weight gain following HFD exposure (Figure S2B). In parallel, HFD feeding resulted in increased subcutaneous and visceral adipose tissue mass, confirming the development of diet‐induced obesity (Figure S2C,D).

Micro‐CT analysis revealed that Firmicutes‐colonized mice displayed structural skeletal adaptation consistent with that observed in Oligo‐MM12‐colonized mice, particularly with respect to increased trabecular mean area under HFD conditions (Figure 2F). In addition, cortical polar moment of inertia and cortical thickness were increased to levels comparable to those observed in Oligo‐MM^12^‐colonized mice (Figure 2G,H, Figure S2E–I).

Together, these findings indicate that even a reduced microbial community dominated by Firmicutes is sufficient to support skeletal adaptation during diet‐induced obesity.

## 4 Discussion

This study identifies the gut microbiota as a critical determinant of bone modeling during diet‐induced obesity. By directly comparing germ‐free and gnotobiotic mice colonized with the defined Oligo‐Mouse‐Microbiota (Oligo‐MM^12^), we demonstrate that microbial colonization enables skeletal adaptation in association with distinct systemic metabolic profiles and altered bone turnover. Colonized mice displayed increased cortical thickness and trabecular area in response to high‐fat diet (HFD) feeding, while germ‐free (GF) counterparts failed to remodel their skeleton despite comparable weight gain. These findings establish a link between microbial colonization and skeletal adaptation under metabolic stress.

The observed increase in trabecular mean area and cortical polar moment of inertia in colonized mice suggests that the microbiota facilitates biomechanical and metabolic adaptation of bone to increased body weight. In contrast, GF animals showed a blunted response and even reduced cortical parameters, indicating that microbial signals are required to sense or translate systemic metabolic cues into local remodeling processes. This microbiota‐dependent coupling of metabolism and bone structure extends earlier observations that GF mice exhibit increased bone mass but reduced bone turnover [16, 19]. Our data further suggest that the microbiota does not simply affect bone mass at baseline but actively regulates adaptive remodeling in response to environmental challenges such as HFD‐induced obesity.

One potential alternative explanation for the observed skeletal differences is increased mechanical loading associated with higher body mass in Oligo‐MM^12^‐HFD mice compared to GF‐HFD mice. Mechanical loading is a well‐established determinant of bone remodeling, and increased body weight has been shown to influence bone mass, architecture, and turnover [3]. In addition, high‐fat diet–induced obesity has been reported to modulate skeletal parameters in murine models [9, 10]. Therefore, the slightly higher absolute body weight observed in Oligo‐MM^12^‐HFD mice may have contributed to the enhanced cortical and trabecular parameters. Although both microbiota groups exhibited comparable weight gain trajectories under HFD feeding, Oligo‐MM^12^ mice reached modestly higher final body weights. Thus, we cannot exclude that mechanical forces partially contributed to the observed skeletal adaptations. However, several findings support an additional microbiota‐dependent component. First, metabolomic differences were evident despite broadly similar weight gain patterns. Second, bone turnover markers differed between microbiota conditions. Third, quantification of subcutaneous and visceral fat depots confirmed comparable obesity development in both groups. Together, these data suggest that both mechanical loading and microbiota‐dependent metabolic remodeling likely contribute to skeletal adaptation under HFD conditions.

Importantly, these findings suggest that skeletal adaptation is not solely determined by mechanical loading or endocrine factors but is also influenced by microbial metabolic activity. This positions the microbiota as an important modulator linking systemic metabolism with local bone dynamics, providing new insight into the gut–bone axis under metabolic stress.

In addition, because total body weight alone may not fully reflect adiposity, we quantified subcutaneous and visceral fat depots at harvest. Both GF and Oligo‐MM^12^ mice exhibited marked increases in adipose tissue mass under HFD conditions, consistent with the observed body weight gain. These findings confirm that both microbiota groups developed comparable diet‐induced obesity, suggesting that differences in skeletal remodeling cannot be solely attributed to disparities in overall adiposity.

Notably, while BV/TV remained unchanged across all groups, differences in trabecular mean area were evident. Increased trabecular mean area with constant BV/TV in Oligo‐MM^12^ ‐HFD mice, corresponds to an increase in the overall bone mass and mechanical competence of the tibia, which reflects the similar formation and lower resorption markers when compared to GF mice. This observation is in line with previous reports showing that BV/TV, while widely used in both preclinical and clinical settings, can remain unchanged despite changes in trabecular morphology and connectivity [35, 36, 37, 38, 39]. Indeed, bones with comparable BV/TV may differ in trabecular thickness, number, and spacing [40]. Consistent with these systemic changes, histological analyses revealed a trend toward more numerous TRAP‐positive osteoclasts in GF HFD mice, whereas colonized HFD‐fed mice displayed fewer osteoclasts but marked accumulation of marrow adipocytes, consistent with reports that bone marrow adipose tissue expands in obesity and acts as an active regulator of bone and energy metabolism [41], and with previous findings that the microbiota modulates osteoclast activity and protects against bone loss under metabolic or hormonal stress [42]. Interestingly, GF HFD mice also exhibited structural alterations in the marrow cavity without clear adipocyte formation, suggesting that distinct remodeling pathways may be engaged in the absence of microbial signals. This observation aligns with human data indicating that increased marrow fat often parallels reduced osteoblast function and bone fragility [43].

Several mechanisms may explain the microbiota's influence on bone remodeling. Microbial metabolites such as SCFAs are known to regulate osteoclast activity, promote calcium absorption, and modulate systemic inflammation thereby influencing bone remodeling [16, 20]. Moreover, GF mice have been reported to exhibit reduced serum calcium and phosphate levels [19], which may impair mineralization and osteoblast function. The suppression of serum β‐CTX levels in colonized mice, independent of diet, further supports a role for the microbiota in restraining bone resorption, in line with prior reports linking dysbiosis to increased osteoclastic activity [18].

Despite elevated circulating levels of several metabolites in GF mice, including lysine, uridine, DHA, linoleic acid, and pyruvate, these animals failed to undergo structural adaptation of trabecular and cortical compartments. Lysine is required for collagen cross‐linking and extracellular matrix formation, whereas uridine has been implicated in osteoblast differentiation and mineralization [21, 44]. Similarly, DHA and linoleic acid can influence inflammatory signaling pathways involved in osteoclast activity and bone resorption [45]. The accumulation of these metabolites in GF mice, together with unchanged P1NP and elevated β‐CTX levels, may therefore reflect altered anabolic utilization combined with sustained resorptive activity. In contrast, ribose levels were higher in colonized animals, which are consistent with increased nucleotide synthesis and cellular proliferation [46]. This interpretation aligns with the reduced β‐CTX levels observed in colonized mice and supports a microbiota‐dependent shift toward balanced bone remodeling.

Together, these findings support a role for microbiota‐dependent metabolic processes in coordinating skeletal adaptation under metabolic stress.

The observation that a reduced Firmicutes‐containing consortium was sufficient to recapitulate key aspects of the skeletal phenotype further supports a microbiota‐dependent mechanism. Firmicutes‐colonized mice exhibited trabecular and cortical adaptations comparable to those observed in Oligo‐MM^12^ animals, despite the markedly reduced microbial complexity. This finding suggests that microbiota‐dependent bone remodeling does not require the full diversity of the Oligo‐MM^12^ community and may instead be driven by functional properties shared within a simplified microbial ecosystem. These data refine the causal interpretation of our results and support a systems‐level mechanism linking microbial colonization to host skeletal adaptation.

One limitation of our study is the relatively short duration of HFD feeding (8 weeks). While sufficient to induce obesity and early skeletal changes, longer interventions may be required to uncover delayed or cumulative effects, especially in cortical bone. A second consideration relates to the use of GF mice as a comparator group. Lifelong absence of microbial exposure is known to result in developmental alterations in immune and metabolic systems [11, 15, 47]. Early‐life microbial colonization plays a critical role in shaping host physiology, and GF animals may therefore exhibit persistent immunological and metabolic differences compared to colonized counterparts [48]. In the skeletal system, GF mice have previously been reported to display altered bone mass and turnover [16, 19]. Although Oligo‐MM^12^ mice in our study were colonized from birth via vertical transmission and thus do not represent adult recolonization models, differences between GF and colonized animals may partially reflect developmental adaptations inherent to lifelong GF physiology.

In addition, the use of the Oligo‐MM^12^ consortium also represents a reductionist approach. While the defined 12‐member community enhances experimental reproducibility and enables mechanistic investigation [22, 23], simplified gnotobiotic communities do not fully recapitulate the ecological complexity and functional diversity of conventional specific pathogen‐free (SPF) microbiota [49]. Consequently, the observed phenotypes may not entirely reflect microbiota–bone interactions within more complex microbial ecosystems.

Moreover, histological observations were not quantified. While they clearly highlight differences in osteoclast abundance and marrow adiposity between groups, quantitative assessment would strengthen these findings. Future work using metabolomics and targeted microbial manipulation will be needed to identify the causal pathways mediating microbiota‐bone crosstalk under metabolic stress.

Finally, our study does not dissect the specific microbial taxa, strain‐derived metabolites, or host molecular pathways responsible for the observed phenotypes. Although the Oligo‐MM^12^ model provides a controlled and reproducible microbial ecosystem, the precise contributions of individual strains and their functional interactions with host metabolic or immune signaling remain to be defined. Future studies employing strain‐specific depletion or mono‐association approaches, targeted metabolomic validation, and genetic manipulation of host pathways will be required to elucidate the mechanisms by which microbial signals regulate bone remodeling under obesogenic conditions. Together, the present findings establish a physiological framework that will guide such mechanistic investigations. While molecular mechanisms remain to be defined, the combination of gnotobiotic modeling, metabolomic profiling, and independent validation in a reduced consortium establishes a causal systems‐level framework linking microbiota composition to skeletal adaptation.

In summary, our findings reveal that the gut microbiota plays a role in skeletal adaptation to diet‐induced obesity. These results have implications for understanding bone fragility in metabolic disease and suggest that microbial‐targeted interventions may hold therapeutic potential to preserve bone health in obesity.

## Author Contributions

M.C.S. and M.L.B. conceived and designed the study. M.C.S., J.B., O.T., T.H., and S.G. performed experiments. M.S. and P.Z. contributed to micro‐CT acquisition and analysis. N.S. and B.G. performed histology analyses. M.C.S. and M.S. analyzed the data. M.C.S. and M.L.B. wrote the manuscript with input from all authors. All authors reviewed and approved the final version of the manuscript.

## Funding

This work was supported by the Swiss National Science Foundation (SNSF Grant PCEFP3_194618/1) and the Pierre Mercier Foundation.

## Ethics Statement

All animal procedures were approved by the Cantonal Veterinary Office of Bern, Switzerland (approval number: BE 48/2022) and were conducted in accordance with the Swiss Animal Welfare Act and institutional guidelines.

## Consent

The authors have nothing to report.

## Conflicts of Interest

The authors declare no conflicts of interest.

## Supporting information


**Figure S1:** Microbiota composition, adiposity, and selected serum metabolites in GF and Oligo‐MM12 mice. (A) Relative abundance of the 12‐member Oligo‐MM^12^ consortium in individual colonized mice determined by 16S rRNA gene sequencing of cecal contents, confirming stable engraftment of all strains. (B) Average daily food intake in germ‐free (GF) and Oligo‐MM^12^ mice fed low‐fat diet (LFD) or high‐fat diet (HFD). (C) Subcutaneous adipose tissue and (D) visceral adipose tissue weight measured at harvest after 8 weeks of dietary intervention. (E–J) Relative abundance of selected serum metabolites identified by untargeted metabolomics comparing GF and Oligo‐MM^12^ mice under HFD conditions: (E) lysine, (F) docosahexaenoic acid (DHA), (G) linoleic acid, (H) uridine, (I) pyruvate, and (J) ribose.
Data are presented as mean ± SD with individual data points shown. Statistical analysis: two‐way ANOVA with Šídák's multiple comparisons test for panels (B–D); unpaired two‐tailed *t*‐tests for panels (E–J). *p < 0.05, **p < 0.01, ***p < 0.001, ****p < 0.0001. *n* = 5–6 per group.

**Figure S2:** Reduced Firmicutes consortium supports skeletal adaptation to diet‐induced obesity. (A) Relative abundance of the five‐member Firmicutes consortium ( *Enterococcus faecalis* KB1, *Flavonifractor plautii* YL31, *Enterocloster clostridioformis* YL32, *Clostridium innocuum* I46, and *Blautia pseudococcoides* YL58) in individual mice determined by 16S rRNA gene sequencing of cecal contents. (B) Final body weight of Firmicutes‐colonized mice fed low‐fat diet (LFD) or high‐fat diet (HFD). (C) Subcutaneous adipose tissue and (D) visceral adipose tissue weight measured at harvest. (E–I) Micro‐CT‐derived trabecular and cortical bone parameters in Firmicutes‐colonized mice: (E) trabecular thickness (Tb.Th), (F) trabecular separation (Tb.Sp), (G) trabecular number (Tb.N), (H) bone volume fraction (BV/TV), and (I) tibial length. Data are presented as mean ± SD with individual data points shown. Statistical analysis: unpaired two‐tailed *t*‐tests were performed for comparisons between LFD and HFD groups. *p < 0.05, **p < 0.01, ***p < 0.001, ****p < 0.0001. *n* = 5 per group.

## Data Availability

Serum Metabolomics Data have been deposited in the MetaboLights repository under accession number MTBLS13384 and are publicly available at https://www.ebi.ac.uk/metabolights/editor/MTBLS13384.
