# Supplementary figures and images for: Defined Microbiota Modulates Host Metabolome and Skeletal Adaptation to Diet‐Induced Obesity
Source: FASEB J. 2026 Apr 21;40:e71831. doi: 10.1096/fj.202600564RR (PMC13098627; doi:10.1096/fj.202600564RR)

Supplementary Figure 1

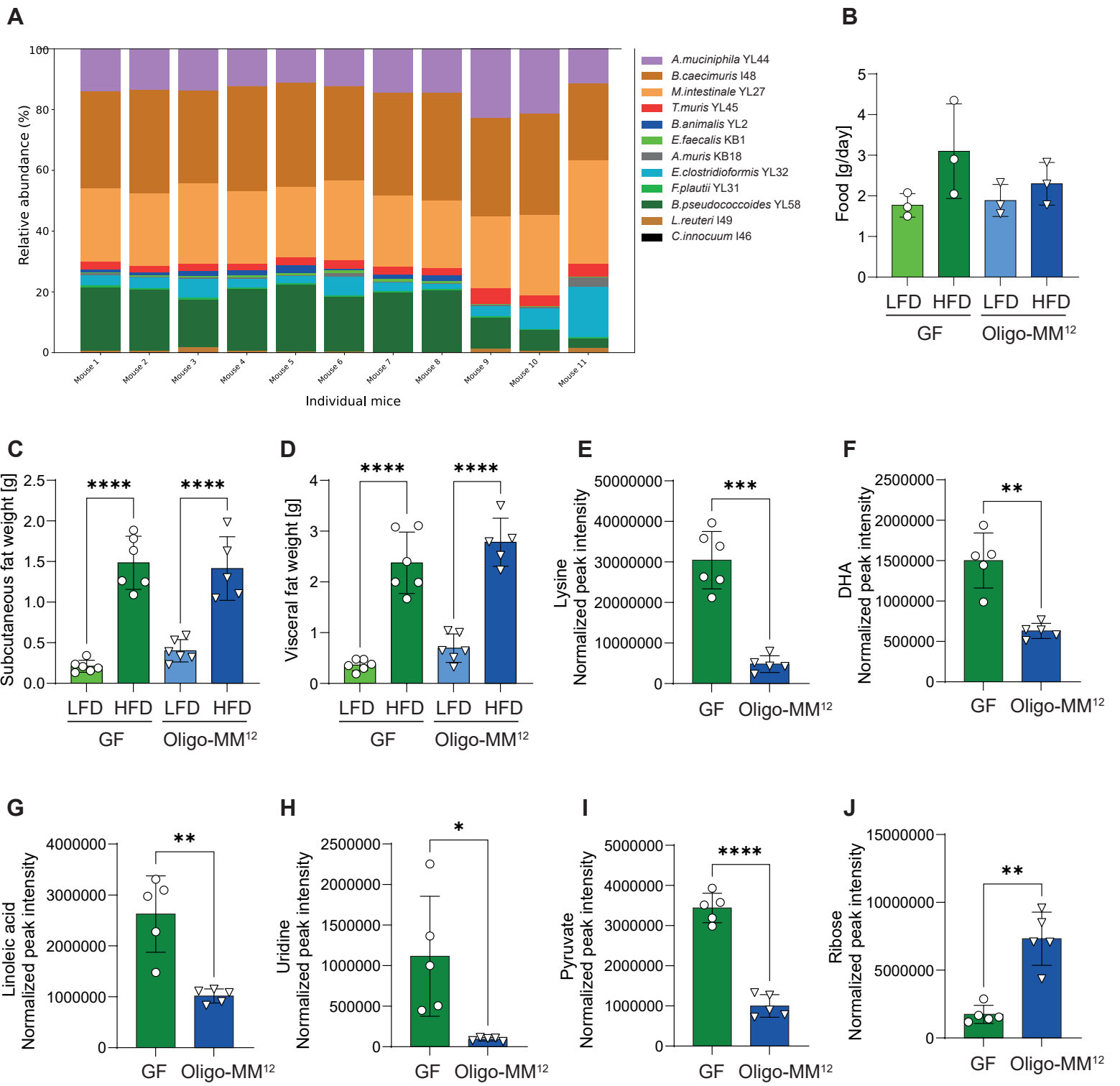

Supplement: Supplementary file 1 — Figure S1: Microbiota composition, adiposity, and selected serum metabolites in GF and Oligo‐MM12 mice. (A) Relative abundance of the 12‐member Oligo‐MM12 consortium in individual colonized mice determined by 16S rRNA gene sequencing of cecal contents, confirming stable engraftment of all strains. (B) Average daily food intake in germ‐free (GF) and Oligo‐MM12 mice fed low‐fat diet (LFD) or high‐fat diet (HFD). (C) Subcutaneous adipose tissue and (D) visceral adipose tissue weight measured at harvest after 8 weeks of dietary intervention. (E–J) Relative abundance of selected serum metabolites identified by untargeted metabolomics comparing GF and Oligo‐MM12 mice under HFD conditions: (E) lysine, (F) docosahexaenoic acid (DHA), (G) linoleic acid, (H) uridine, (I) pyruvate, and (J) ribose. Data are presented as mean ± SD with individual data points shown. Statistical analysis: two‐way ANOVA with Šídák's multiple comparisons test for panels (B–D); unpaired two‐tailed t‐tests for panels (E–J). *p < 0.05, **p < 0.01, ***p < 0.001, ****p < 0.0001. n = 5–6 per group. [file FSB2-40-e71831-s001.pdf]

Supplementary Figure 2

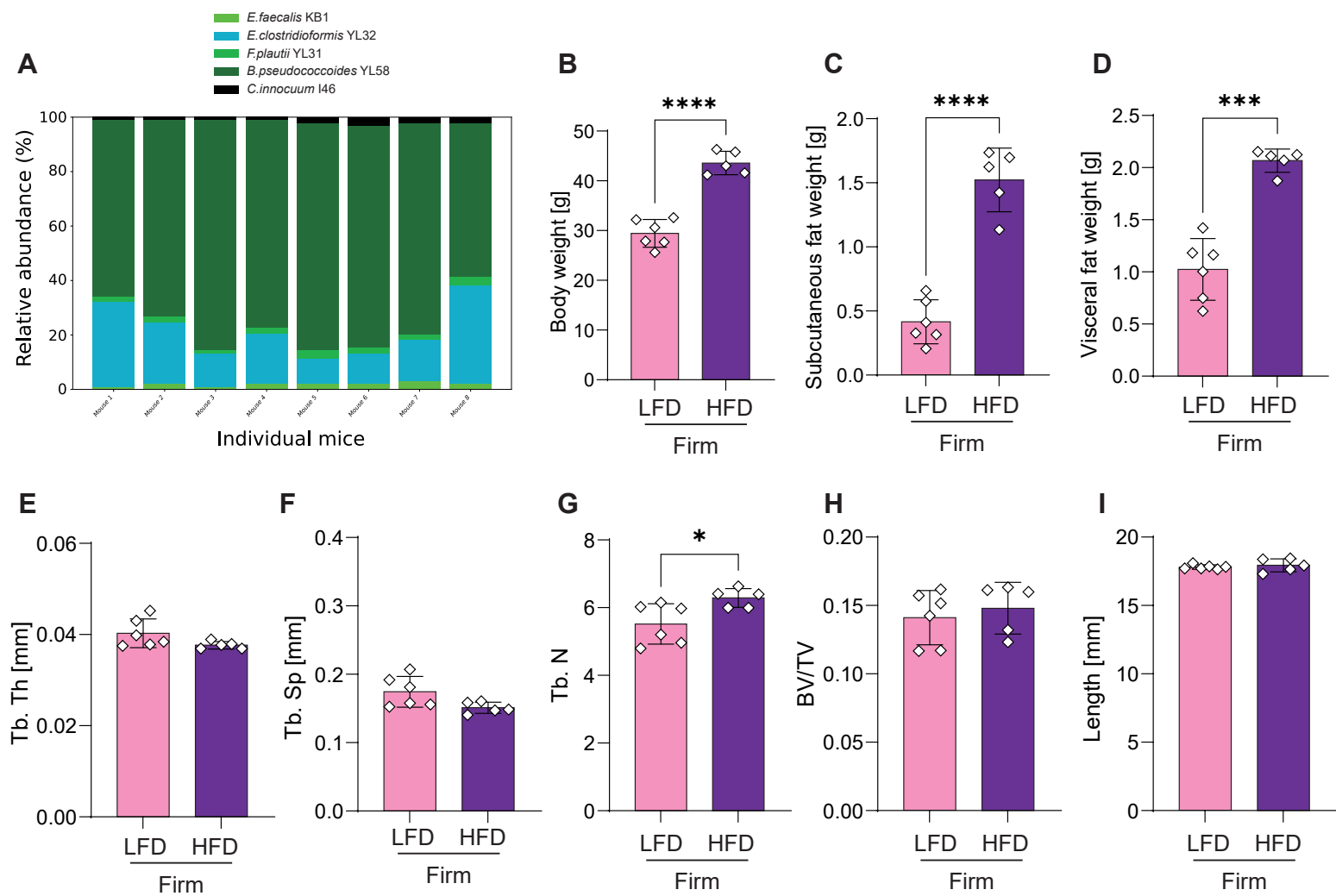

Supplement: Supplementary file 2 — Figure S2: Reduced Firmicutes consortium supports skeletal adaptation to diet‐induced obesity. (A) Relative abundance of the five‐member Firmicutes consortium ( Enterococcus faecalis KB1, Flavonifractor plautii YL31, Enterocloster clostridioformis YL32, Clostridium innocuum I46, and Blautia pseudococcoides YL58) in individual mice determined by 16S rRNA gene sequencing of cecal contents. (B) Final body weight of Firmicutes‐colonized mice fed low‐fat diet (LFD) or high‐fat diet (HFD). (C) Subcutaneous adipose tissue and (D) visceral adipose tissue weight measured at harvest. (E–I) Micro‐CT‐derived trabecular and cortical bone parameters in Firmicutes‐colonized mice: (E) trabecular thickness (Tb.Th), (F) trabecular separation (Tb.Sp), (G) trabecular number (Tb.N), (H) bone volume fraction (BV/TV), and (I) tibial length. Data are presented as mean ± SD with individual data points shown. Statistical analysis: unpaired two‐tailed t‐tests were performed for comparisons between LFD and HFD groups. *p < 0.05, **p < 0.01, ***p < 0.001, ****p < 0.0001. n = 5 per group. [file FSB2-40-e71831-s002.pdf]
